# Supplementary figures and images for: Evaluation method for cell-free in situ tissue-engineered vasculature monitoring: Proof of growth and development in a canine IVC model
Source: PLoS One. 2022 Apr 18;17(4):e0267274. doi: 10.1371/journal.pone.0267274 (PMC9015146; doi:10.1371/journal.pone.0267274)

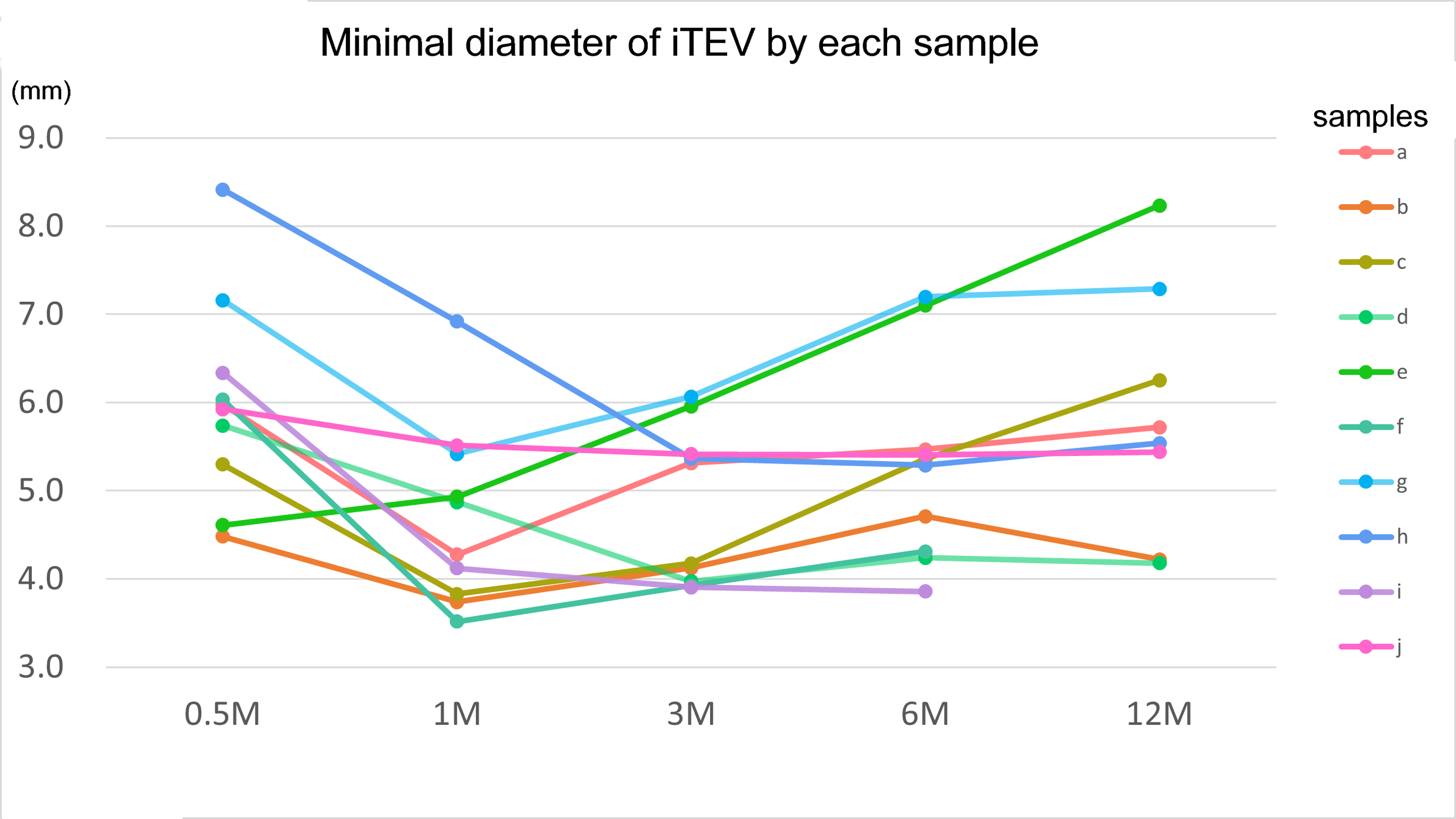

Supplement: S1 Fig — Time-course changes in minimal diameter of iTEV by sample are shown. (TIF) [file pone.0267274.s001.tif]

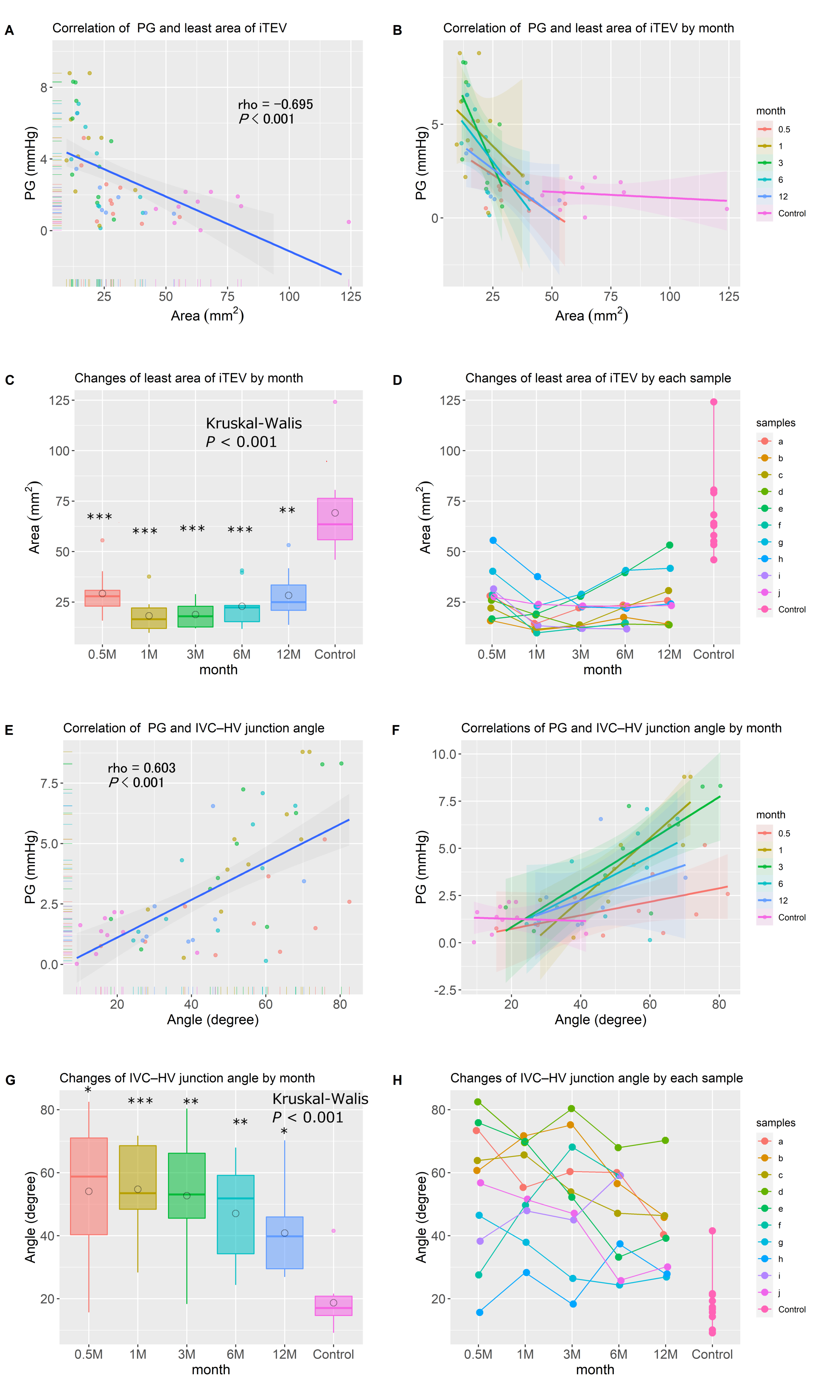

Supplement: S2 Fig — (A) Correlation between the least estimated area of iTEV and pressure gradient. Spearman’s rank correlation Rho value of the HV index is shown. Rho = -0.695 (95% confidence interval: -0.792, -0.540; P < 0.001). (B) Correlation between the least estimated area and pressure gradient by month (Estimated Rho = -0.542). In native IVC, Rho = 0.188, and in iTEV at 0.5 months, Rho = -0.636; at 1 month, Rho = -0.261; at 3 months, Rho = -0.503; at 6 months, Rho = -0.770; and at 12 months, Rho = -0.904. There was no difference in the correlation between months (P = 0.060, NS: not significant). (C) Changes in the least estimated area of iTEV by month expressed as box and whisker plots. Lines represent the lower, median, and upper quartile values. Whiskers represent the extent of the remaining data. Circles represent mean values. The least estimated area, P < 0.001 using the Kruskal–Wallis test. The Steel–Dwass test was used for posthoc analysis. Native vs. 0.5 month (*, P < 0.001); native vs. 1 month (***, P < 0.001); native vs. 3 months (***, P < 0.001); native vs. 6 months (***, P < 0.001); and native vs. 12 months (***, P = 0.0016). (D) Each dot-line graph shows time-dependent changes in the least estimate area by sample. (E) Correlation between the IVC-HV junction angle and pressure gradient. Spearman’s rank correlation Rho value of the HV index is shown. Rho = 0.603 (95% confidence interval: 0.405, 0.757; P < 0.001). (F) Correlation between the IVC-HV junction angle and pressure gradient by month (Estimated Rho = 0.638). In native IVC, Rho = 0.285, and in iTEV at 0.5 months, Rho = 0.503; at 1 month, Rho = 0.903; at 3 months, Rho = 0.770; at 6 months, Rho = 0.455; and at 12 months, Rho = 0.571. There was no difference in the correlation between months (P = 0.254, NS: not significant). (G) Changes in the IVC-HV junction angle of iTEV by month expressed as box and whisker plots. Lines represent the lower, median, and upper quartile values. Whiskers represent the extent of the [file pone.0267274.s002.tif]

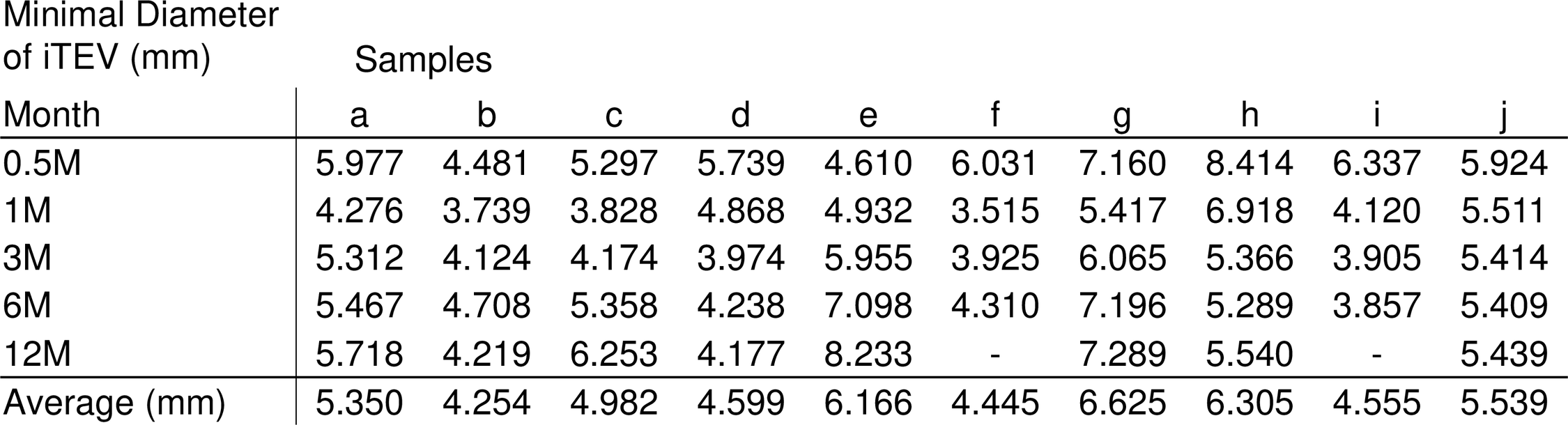

Supplement: S1 Table — Raw data of time-course changes in minimal diameter of iTEV by dample are shown. (TIF) [file pone.0267274.s003.tif]
